# Current News and Opinion

**Published:** 1883-08

**Authors:** 


### Current News and Opinion.

### THE DENTAL CALENDAR.

THE AMERICAN DENTAL ASSOCIATION meets annually the first Tuesday in August.

W. H. GODDARD, President.
Louisville, Ky.
GEO. H. CUSHING, Secretary,
34 Monroe Street, Chicago.

THE AMERICAN DENTAL CONVENTION meets annually at Saratoga, on the second Tuesday of August.

F. Y. CLARK, President.
A. C. RICH, Secretary,
Saratoga, N. Y.

THE NEW YORK ODONTOLOGICAL SOCIETY meets on the third Tuesday of each month, at residence of members.

S. G. PERRY, President.
E. T. PAYNE, Rec. Sec.
J. MORGAN HOWE, Cor. Sec.
34 W. 35th St., N. Y. City.

THE ODONTOLOGICAL SOCIETY OF PHILADELPHIA meets on the last Saturday of each month, at residence of members.

JAMES TRUMAN, President.
S. H. GUILFORD, Cor. Secretary.
AMBLER TEES, Rec. Secretary,
548 North Seventeenth Street.

THE ODONTOGRAPHIC SOCIETY OF PHILADELPHIA meets on the first Monday in each month, at residence of members.

L. ASHLEY FAUGHT, President.
G. F. WYMAN, Cor. Secretary.
ALONZO BOICE, Rec. Secretary,
Vine Street, corner Sixteenth.

The Pennsylvania Association of Dental Surgeons (Philadelphia) meets the first Tuesday in each month at Penna. Dental College.

J. H. Gilherns, President.

T. F. Chupein, Rec. Secretary,

1408 Pine Street.

The American Academy of Dental Science (Boston), meets on the first Wednesday of each month, at residence of members.

G. T. Moffatt, President.
H. F. Hamilton, Secretary.

The Society for Advancement of Oral Science (Boston), meets the last Thursday in December, March, June, and September, at residence of members.

D. M. Parker, President.
W. H. Rollins, Secretary.

The Harvard Odontological Society meets the last Thursday in each month, at residence of members.

J. G. W. Werner, President.
A. J. Colgan, Secretary.

The Harvard Dental Alumni Association meets annually at Young's Hotel, Boston, on the Tuesday evening preceding commencement.

F. E. Bamfield, President.

T. O. Loveland, Secretary.

The Dental Society of the State of New York meets annually at Albany, on the second Wednesday in May.

L. S. Straw, President.
J. Edward Line, Secretary,
Rochester.

The First District Dental Society meets at the S. S. White Dental Mfg. Co.'s rooms, corner Broadway and Thirty-second Street, on the first Tuesday evening of each month. Clinics at the S. S. White Dental Mfg. Co.'s rooms, corner Broadway and Ninth Street, in the afternoon of every meeting day.

A. L. Northrop, President.
James E. Dexter, Secretary,
8 East Thirty-fourth Street, N. Y. City.

The Second District Dental Society meets on the first Tuesday in March, June, September, and December, at the residence of members.

E. H. Dickey, President.
J. J. Pitts, Secretary,
191 Clinton Street, Brooklyn.

The Third District Dental Society meets annually on the third Tuesday in April, at place appointed at the previous meeting.

S. H. Welch, President.
A. M. Wright, Secretary,
38 Third Street, Troy, N. Y.

The Fourth District Dental Society meets annually on the second Wednesday in August, at Saratoga Springs.

W. H. Colgrove, President.
A. C. Rich, Secretary,
Saratoga Springs.

The Fifth District Dental Society holds its annual meeting at Syracuse, the second Tuesday in April.

G. V. N. Relyea, President.
G. S. Curtiss, Secretary.
Syracuse.

The Sixth District Dental Society holds its annual meeting in Binghamton, the first Tuesday in May.

W. D. Jewell, President.
E. D. Downs, Secretary.
Owego.

The Seventh District Dental Society holds its annual meeting in Rochester, the last Tuesday in April.

F. E. Howard, President.
J. S. Walter, Secretary.
Rochester.

THE EIGHTH DISTRICT DENTAL SOCIETY holds its annual meeting in Buffalo, on the third Tuesday in April.

S. A. FREEMAN, President.
C. S. BUTLER, Secretary,
Buffalo.

The Seventh and Eighth District Societies hold a semi-annual union meeting on the last Tuesday of October. On the even year in Rochester, odd year in Buffalo.

THE PENNSYLVANIA STATE DENTAL SOCIETY meets annually on the last Tuesday in July, at such place as may be designated at the previous annual meeting.

J. C. GREEN, President.

E. P. KREMER, Rec. Sec.

W. H. FUNDENBERG, Cor. Sec.

330 Penn. Avenue, Pittsburgh.

THE NEW JERSEY STATE DENTAL SOCIETY meets annually, on the third Wednesday in July. Meeting of 1884 will be held at Asbury Park.

E. H. BUNTING, President.
CHAS. E. MEEKER, Secretary,
27 Fulton Street, Newark, N. J.

THE MASSACHUSETTS DENTAL SOCIETY holds its annual meeting at Boston, on the second Thursday in December, and its semi-annual meeting at such place as may be designated at the annual meeting.

F. SEARL, President.
W. E. PAGE, Secretary,
110 Tremont Street, Boston.

THE CENTRAL DENTAL SOCIETY OF NORTHERN NEW JERSEY meets the last Thursday in each month, at residence of members.

F. W. LEVY, President,
J. G. PALMER, Secretary,
New Brunswick.

THE BROOKLYN DENTAL SOCIETY meets on the second Monday of each month, at the residence of members.

A. H. BROCKWAY, President.
C. P. CRANDELL, Secretary,
508 Clinton Avenue.

THE PITTSBURGH, PA., DENTAL ASSOCIATION meets on the first Tuesday in each month, at residence of members.

F. A. REINHART, President.
W. H. FUNDENBERG, Secretary,
330 Pennsylvania Avenue, Pittsburgh, Pa.

THE ROCHESTER DENTAL CLUB meets on the first Wednesday evening in each month, except July, August and September.

F. D. BROWNE, President.
R. SALTER, Secretary,
Rochester.

---

### AMERICAN DENTAL ASSOCIATION.

The twenty-third annual meeting of the American Dental Association will take place at Niagara Falls, commencing Tuesday, August 7, 1883, at 10 A. M.

GEO. H. CUSHING,
Recording Secretary.

The Committee on Credentials and the Treasurer will be at the place of meeting at 8 A. M., Tuesday, at which time it is hoped the members and delegates will present their credentials and pay their dues, as far as possible, before the hour for the regular meeting.

The afternoon of Tuesday will be set apart for the meeting of the different Sections, to enable them to complete their reports to be presented to the general association.

J. N. CROUSE,
Chairman Executive Committee.

---

### AMERICAN DENTAL CONVENTION.

The American Dental Convention will hold its next annual meeting at Saratoga Springs, on the second Tuesday in August, 1883. A large attendance is expected, and quite a number of interesting papers are promised.

A. C. RICH, Secretary,
Saratoga Springs, N. Y.

NOTICE TO STATE BOARDS OF DENTAL EXAMINERS.

There will be held at the Cataract House, Niagara Falls, on Monday, August 6, 1883, at 2 o'clock P. M., a meeting of all the State Boards of Dental Examiners, for the purpose of perfecting the organization of a National Association of Examining Boards. It is hoped that every Board will be fully represented.

GEO. H. CUSHING,
Secretary of Conference held at Lexington, Ky.

---

THE sixth annual meeting of the American Society of Microscopists will be held in the city of Chicago, beginning Tuesday, August 7, 1883, and continuing four days. Ample preparations are being made by the Committee of the State Microscopical Society of Illinois, and the Chicago Academy of Sciences, and the attendance of members, and those desiring to become members, is expected to be larger than ever before. First-class hotel accommodations at reduced special rates have been secured, and choice arrangements made for the comfort and convenience of the meeting.

ALBERT MCCALLA, A. M., President,
Fairfield, Iowa.

D. S. KELLICOTT, PH. D., Secretary,
Buffalo, N. Y.

---

BLESSED AMERICA.

The proportion of doctors to population is given as follows by the *Siglo-medico* :

| | |
|---|---|
| France, - - - - | 2.91 per 10,000. |
| Germany, - - - - | 3.21 " |
| Austria, - - - - | 3.41 " |
| England, - - - - | 6 " |
| Hungary, - - - - | 6.10 " |
| Italy, - - - - | 6.10 " |
| Switzerland, - - - | 7.06 " |
| United States, - - - | 16.24 " |

### HONOR TO A DENTIST.

Edwin Saunders, of London, England, for thirty-seven years dentist to the Queen, has been knighted, and will henceforth be known as Sir Edwin Saunders. As the President of the Dental Section of the International Congress of 1881, Mr. Saunders was called upon to extend the hospitalities of the profession in England to visiting guests, and he accomplished the task in a peculiarly graceful and happy manner.

For many years he has been intimately connected with the cause of dental education, and has been its generous patron. He was a distinguished and skillful practitioner, and these were reasons quite sufficient for the unusual honor so worthily bestowed, aside from the fact of his being dentist to the Queen, which in England is a qualification of itself.

